# Movement Disorders in COVID-19: Whither Art Thou?

**DOI:** 10.5334/tohm.553

**Published:** 2020-08-12

**Authors:** Howard L. Geyer, David M. Kaufman, Raminder K. Parihar, Mark F. Mehler

**Affiliations:** 1 Saul R. Korey Department of Neurology, Albert Einstein College of Medicine, Bronx, NY, US; 2 Roslyn and Leslie Goldstein Laboratory for Stem Cell Biology and Regenerative Medicine, Institute for Brain Disorders and Neural Regeneration, Departments of Neuroscience, Psychiatry and Behavioral Sciences, Rose F. Kennedy Center for Research on Intellectual and Developmental Disabilities, Albert Einstein College of Medicine, Bronx, NY, US

**Keywords:** SARS-CoV-2, movement disorders, encephalitis

**Inspector Gregory:** Is there any other point to which you would wish to draw my attention?

**Sherlock Holmes:** To the curious incident of the dog in the night-time.

**Gregory:** The dog did nothing in the night-time.

**Holmes:** That was the curious incident.

Sir Arthur Conan Doyle, “The Adventure of Silver Blaze,” *The Memoirs of Sherlock Holmes*

As neurologists working at a major academic medical center in the Bronx, a region that has been afflicted with an exceptionally high prevalence of COVID-19, particularly among socio-economically disadvantaged populations with a higher incidence and prevalence of SARS-CoV-2/COVID-19 comorbidities, we have seen a diversity of primary and secondary neurologic manifestations of the disease. Our services have been filled with patients who have sustained ischemic stroke, intracerebral hemorrhage, encephalopathies, headache, alterations in circadian rhythm, increased seizure frequency, myopathy, Guillain-Barré syndrome and its Miller Fisher variant, anosmia/ageusia, and other neurological complications, often with atypical presentations. Gastrointestinal dysfunction is very common, likely due to disruption of the enteric nervous system, and the frequently encountered respiratory failure also has been posited to relate to multifaceted nervous system pathology [1]. The range of neurologic dysfunction associated with COVID-19 has been reviewed thoroughly [2]. However, we have been impressed by the paucity of patients presenting with *de novo* movement disorders, both in our center and in the literature. Of 601 COVID-19-positive patients in our hospital system who received a neurologic consultation between March 1 and April 17, 2020, not a single one of these consultations (0%) was requested for a concern pertaining to a movement disorder.

That the novel coronavirus SARS-CoV-2, the organism responsible for COVID-19, would cause neurologic dysfunction is no surprise. Other b-coronaviruses, including SARS-CoV, the virus most similar to SARS-CoV-2, appear to have neuroinvasive potential when studied in transgenic mice [3]. SARS-CoV-2 is thought to enter human host cells via angiotensin-converting enzyme-2 (ACE2) receptors, which are found in upper and lower airways, cardiac, hepatic, renal and gastrointestinal cells, and brainstem nuclei, among other sites. The virus may reach the central nervous system by invading peripheral nerve terminals, such as those in the gastrointestinal tract or nasal cavity, and traveling trans-synaptically to the brain [4]. The virus also may act directly on the brainstem, disrupting respiratory control nuclei in addition to other cardinal homeostatic functions [1].

A prior outbreak due to a coronavirus, with SARS-CoV as the causative organism, occurred in 2002–2004. That epidemic featured severe acute respiratory syndrome but neurologic complications were rare, primarily polyneuropathy, myopathy, and large-vessel ischemic stroke [5]. Moreover, when neurologic complications occurred they developed later in the disease. With COVID-19, in contrast, neurologic dysfunction often predominates and/or is present at onset. What accounts for the discrepancy between these two related viruses in terms of neurologic involvement? One possibility is that SARS-CoV-2 might exhibit a greater degree of neurotropism than its predecessor. Another conceivable explanation is that its higher virulence results in a greater propensity to elicit host responses, such as cytokine release, inflammation, and/or autoimmunity, and also produces more severe morbidities, like hypoxemia, nephropathy, and/or other critical illnesses, which in turn affect the central and peripheral nervous systems. Alternatively, the greater clinical polymorphism is simply due to the far greater prevalence of the current pandemic, with consequently more variability in presentation and a higher regional mutational spectrum.

The paucity of movement disorders associated with COVID-19 is particularly striking when contrasted with the neurologic syndrome which affected over a million people worldwide in the aftermath of the 1918 “Spanish” influenza, termed by Constanin von Economo *encephalitis lethargica*. Whether the flu directly caused the encephalitis or predisposed to its development (perhaps by triggering an autoimmune response targeted against basal ganglia epitopes) remains a matter of debate, but the temporal coincidence suggests an etiologic role for the virus. That encephalitis was associated with a wide range of movement disorders, of which post-encephalitic parkinsonism is the best known, although other manifestations in the acute phase included dystonia, tremor, chorea, myoclonus, and oculomasticatory myorhythmia [6,7]. Although encephalitis has been described as a cardinal neurological manifestation of COVID-19 during the acute phase of illness [8,9], we have yet to encounter any of these associated movement disorder presentations. (In the time since this write-up was first prepared, patients with acute movement disorders and COVID-19 have been reported exiguously; we know of four such reports, which describe myoclonus [10,11], a hypokinetic-rigid syndrome [12], and tremor/ataxia [13]. Encephalitis was present in some of those patients. Whether COVID-19 played a direct role in causing the movement disorders is unproven.)

There are additional reasons to expect that SARS-CoV-2 would give rise to movement disorders. Immunofluorescence studies have shown that dopaminergic neurons as well as microglia and astrocytes bear ACE2 receptors [14]. In a knockout mouse model, deletion of ACE2 receptors resulted in markedly decreased spontaneous global motor activity as well as a significant reduction in striatal dopamine D1 mRNA expression [15]. (Another tantalizing finding of that study is that the substantia nigra of these mice exhibited increased mRNA expression of pro-inflammatory markers including interleukin-6, a cytokine that is elevated in COVID-19 patients and correlates with disease severity [16]). Expression of the gene for ACE2 is coregulated with expression of the gene that codes for dopamine decarboxylase, the enzyme which catalyzes conversion of L-3,4-dihydroxyphenylalanine (L-DOPA) into dopamine; downregulation of ACE2 (which has been shown to occur with SARS-CoV) could thereby alter dopamine synthesis [17]. In the clinical realm, MRI studies of patients with encephalitis have shown a predilection for respiratory viruses to cause abnormalities in the basal ganglia [18]. Older studies have shown affinity of coronaviruses for basal ganglia in mice and monkeys [19], and another study showed increased antibody titers to coronaviruses in the cerebrospinal fluid of patients with Parkinson’s disease, but not in that of healthy control subjects or patients with other neurological disorders [20].

What might account for the dearth of expected movement disorders in COVID-19 patients? Perhaps the damage that SARS-CoV-2 wreaks on the brain, whether direct or indirect, spares the basal ganglia and other areas involved in controlling movement, contrary to expectations. Alternatively, it may be that movement disorders do indeed occur, but other, more severe manifestations (such as paresis or altered consciousness) eclipse them. Another possibility is that patients are not identified because they avoid medical attention for relatively milder symptoms (e.g., due to fear of exposure to infection) while they continue to present for more disabling conditions. It is also likely that rather than an acute process, a delayed and progressive neurodegenerative process will result instead from SARS-CoV-2 infection.

Clearly the unanswered questions far exceed what is known. Progress in virology and other basic sciences will shed light on these issues. For example, immunohistochemical studies further defining the normal modular distribution of ACE2 receptors in the brain and modulation of their expression levels and cellular pliancy following receptor engagement of SARS-CoV2 and its ancillary protein co-regulators, including the spike glycoprotein and associated protease cleavage sites [21], will be invaluable for understanding the cerebral effects of the virus. Whether SARS-CoV-2 affects dopamine synthesis or function should be studied further in the laboratory and in patients. In addition, prospective studies of COVID-19 patients, including detailed clinical descriptions and serial neuroimaging and laboratory data, will help define the natural history of the neurologic aspects of the disease, both the short-term effects as well as the intermediate- and long-term sequelae. Vigilance for post-encephalitic movement disorders will be crucial in the coming years, and a careful history of the patient’s health in 2019–2020 will be critical in assessing patients who subsequently present with neurodegenerative diseases. Large-scale registries will be pivotal for this surveillance [2]; national efforts include those by the Deutsche Gesellschaft für Neurologie (https://www.dgn.org/rubrik-themen/3958-registerstudie-zu-covid-19-der-jungen-neurologen) and the Sociedad Española de Neurología (https://www.sen.es/covid-19). The Movement Disorders Society also hosts a valuable repository of useful and current information (https://www.movementdisorders.org/COVID-19-Pandemic-MDS.htm), including resources for patients.

For the time being, in addition to continuing to provide our patients with optimal care and counseling, we must also respond to the pandemic by educating them about appropriate safety measures, directing them to reliable sources of information, and alleviating their justifiable anxieties. By doing so we can keep our patients as healthy as possible through the crisis and beyond.
